# Plant-Pathogen Interaction

**DOI:** 10.3390/biology10050444

**Published:** 2021-05-19

**Authors:** Maria Doroteia Campos, Mariana Patanita, Carla Varanda, Patrick Materatski, Maria do Rosário Félix

**Affiliations:** 1 MED—Mediterranean Institute for Agriculture, Environment and Development, Instituto de Investigação e Formação Avançada, Universidade de Évora, Pólo da Mitra, Ap. 94, 7006-554 Évora, Portugal; mpatanita@uevora.pt (M.P.); carlavaranda@uevora.pt (C.V.); pmateratski@uevora.pt (P.M.); 2 MED—Mediterranean Institute for Agriculture, Environment and Development & Departamento de Fitotecnia, Escola de Ciências e Tecnologia, Universidade de Évora, Pólo da Mitra, Ap. 94, 7006-554 Évora, Portugal; mrff@uevora.pt

Plant diseases result in severe losses to natural plant systems, and also cause problems for economics and production in agricultural systems. While many biotic constraints are well known and confronted with variable success, the occurrence of emerging pathogens and the progressive incidence of novel virulent strains, races, or pathotypes is evident. Plant disease management faces challenges due to the increasing incidence of emergent diseases, with a consequent decrease in the production potential of agriculture. Furthermore, the deteriorating ecology of agro-ecosystems and the depletion of natural resources, together with an increased risk of disease epidemics resulting from agricultural intensification and monocultures, should be taken into account. Moreover, the practicability of some of the currently available plant protection measures is questionable. The UE directories for commercialization withdrawal of several chemical substances used for pest and disease control, and the new rules for reducing agricultural greenhouse gas emissions contained in the 2015 Paris Agreement of the United Nations Framework Convention on Climate Change, bring new challenges to agricultural production.

In face of this, new strategies of control are needed and an understanding of how pathogens adopt and appropriate adaptive mechanisms during plant infection, and the exploitation of the diversity of mechanisms that plants possess to control their resistance/susceptibility to plant diseases, will aid in nature conservation and ecosystem services, and is also of benefit to agriculture and forestry.

Plants responses to biotic stresses are very complex, as the multitude of interactions involves two living organisms; the plant and the pathogen. Knowledge about plant–pathogen interactions may aid in the prevention of disease in plants. From a coevolutionary perspective, plants recognize and respond to pathogens in several phases. In general, the interaction starts with the plants evolving a multilayered immune system that prevents or hinders colonization by most potential pathogens [1]. This stage of defense is triggered by a class of immune receptors upon recognition of pathogen-associated molecular patterns, perceived by pattern recognition receptors [2,3]. This prompted plants, in turn, to develop specific resistant (R)-proteins that recognize the pathogen/pest effector(s) and initiate an immune mechanism termed effector-triggered immunity [4]. The mechanism implies, after the initial recognition, the induction by the plant of acquired resistance while the pathogen tries to counteract the plant’s resistance.

Advances in plant pathology and biotechnology have allowed an increase in the resistance of crops and the deployment of biological control measures, supported by the enormous growth in information about plant hosts and pathogens at the cellular and molecular levels that has occurred over recent years. New molecular tools, such as next-generation sequencing and clustered regularly interspaced short palindromic repeats (CRISPR) technologies, have greatly accelerated research in biological sciences and offer great opportunities to better understand the molecular networks of plant–pathogen interactions that might aid in the development of innovative genetic engineering strategies, so as to ensure food safety and sustainable agricultural development in the future. Moreover, the identification of regulatory components involved in protection against pathogens is therefore of major importance to a new concept of plant disease management, namely relying on the plant’s innate immune mechanisms.

In this context, this Special Issue brings new experimental results and some advances that may help us to unravel the complexity of plant–pathogen interactions.

## Data Availability

Not applicable.
